# Novel and Future Treatment Options in Mesothelioma: A Systematic Review

**DOI:** 10.3390/ijms23041975

**Published:** 2022-02-10

**Authors:** Danijela Štrbac, Vita Dolžan

**Affiliations:** 1 Institute of Oncology, Zaloška 2, 1000 Ljubljana, Slovenia; dstrbac@onko-i.si; 2 Pharmacogenetics Laboratory, Institute of Biochemistry and Molecular Genetics, Faculty of Medicine, University of Ljubljana, Vrazov trg 2, 1000 Ljubljana, Slovenia

**Keywords:** mesothelioma, chemotherapy, immunotherapy, CAR-T cells, vaccine therapy, gene therapy

## Abstract

Mesothelioma is a rare tumor, frequently associated with asbestos exposure, arising from pleura and peritoneum. Traditionally, diagnosis and treatment have been difficult in a clinical setting. The treatment is based on a trimodal approach involving surgery, chemotherapy, and radiotherapy. The introduction of chemotherapy improved the overall survival. However, the regimen of pemetrexed/cisplatin doublet has not been changed as a standard treatment since 2004. Novel combinations of ipilimumab and nivolumab have only been approved for clinical use in late 2020. The aim of this review was to systematically summarize findings on novel treatment options in mesothelioma. We searched available medical databases online, such as PubMed and Clinicaltrials.gov, to systematically review the literature on novel approaches in immunotherapy, vaccines, and Chimeric Antigen Receptor (CAR)-T cell therapy in mesothelioma. We manually screened 1127 articles on PubMed and 450 trials on ClinicalTrials.gov, and 24 papers and 12 clinical trials published in the last ten years were included in this review. Immunotherapy that was swiftly introduced to treat other thoracic malignancies was slow to reach desirable survival endpoints in mesothelioma, possibly due to limited patient numbers. Novel treatment approaches, such as CAR-T cell therapy, are being investigated. As the incidence of mesothelioma is still rising globally, novel treatment options based on a better understanding of the tumor microenvironment and the genetic drivers that modulate it are needed to support future precision-based therapies.

## 1. Introduction

Mesothelioma is a rare disease with over 30,000 new cases worldwide in 2020. The mortality of this disease was reported for over 26,000 patients, representing 45% of all new cases in Europe in 2020 [1]. The incidence of mesothelioma has risen in recent decades due to asbestos exposure. The latency period from asbestos exposure to disease is over 25 years long [2,3]. However, there is problem of under-reporting in middle and lower income countries, where the rise of mesothelioma incidence is higher than in some developed countries [4].

The pathogenesis of mesothelioma is complex due to the interplay of environmental and genetic factors influencing the inflammatory tumor microenvironment. As recently reviewed by Hiltbrunner et al., mesothelial cells exposed to asbestos fibers secrete C-C chemokine ligand 2 (CCL2), which attracts macrophages. Reactive-oxygen species induced DNA damage, and mutations in mesothelial cells lead to necrotic cell death and the production and release of damage-associated molecular patterns (DAMPs), including High Mobility Group Box 1 protein (HMGB1). The binding of HMGB1 to mesothelial cells enhances their proliferation and migration capacity. Furthermore, the recruitment of macrophages leads to phagocytosis of asbestos fibers, resulting in the secretion of proinflammatory mediators, such as TNF-α, that support carcinogenesis and cancer cells survival. Asbestos fibers and DAMPs also activate inflammasome that activates caspase 1, which activates pro-IL-1β into IL-1β. TNF-α released by macrophages activates the NF-κB signaling pathway in mesothelial cells and supports their survival when exposed to asbestos. Therefore, TNF-α and IL-1β are important players mediating mesothelioma malignant transformation and progression [5].

Besides the crucial role of the inflammatory microenvironment in mesothelioma development and progression, genetic and epigenetic mechanisms have an important role. Oncogene activating mutations are rare, but the loss of function mutations in tumor suppressor genes have a prominent role in mesothelioma. Two of the most frequently mutated tumor suppressor genes in mesothelioma are neurofibromatosis type 2 (NF2) and BRCA1-associated protein-1 (BAP1) genes. NF2 encodes Merlin, which regulates multiple signaling pathways, including the Hippo pathway, and is a critical regulator of contact-dependent inhibition of proliferation and cell growth. BAP1 encodes a nuclear ubiquitin C-terminal hydrolase, one of several classes of deubiquitinating enzymes. BAP1 interacts with several proteins, such as BRCA1 and BARD1, which form a tumor suppressor heterodimeric complex. It also interacts with histone-modifying complexes, such as the Polycomb group repressive deubiquitinase complex.

Furthermore, alterations in DNA methylation patterns have been observed in mesothelioma, especially in E-cadherin, fragile histidine triad, retinoic acid receptor-β, and wnt inhibitory factor-1. Dysregulation of microRNA (miRNA) expression may also contribute to the development of mesothelioma. MiRNAs are short non-coding RNAs that regulate gene expression at the post-transcriptional level. Several miRNAs have been extensively investigated for their potential tumor suppressor role in mesothelioma or as a promising biomarker and possible anti-proliferative and anti-tumor activity. For example, overexpression of hsa-miR-29c* resulted in a significant decrease in proliferation and migration in mesothelioma cell lines. Therefore, overexpression of hsa-miR-29c* could impact a more favorable prognosis in epithelioid mesothelioma patients [5,6]. Furthermore, cell cycle regulation can influence the progression of mesothelioma. A mechanism of aberrant and homozygous deletion status of the p16 *CDKN2A* gene can promote mesothelioma growth since p16 is a protein that slows cell division by slowing the progression of the cell cycle from G1 to S phase [7,8,9].

Mesothelioma can be classified into two main types: pleural and peritoneal mesothelioma; however, there are also rare manifestations of mesothelioma in tunica albuginea of the testis. Histological classification divides mesothelioma into epitheloid, sarcomatoid, and biphasic, but different histological types may overlap [10]. The clinical presentation of mesothelioma can be late in advanced disease with pain, dry cough, weight loss, and fatigue. While asbestos exposure is the leading risk factor in developing mesothelioma, rare cases are genetically predisposed, such as in *BAP1* mutations. Mesothelioma can be staged by tumor, node, metastasis (TNM) classification according to the latest eighth TNM classification edition [11]. Tumors involving the unilateral parietal pleura (T1N0M0) are grouped into stage IA. In contrast, tumors involving visceral pleura and/or mediastinal fat, solitary infusion of the chest wall, and no nodal involvement (T2,3N0M0) are staged as IB. Stage II involves T1,2 N1M0 tumors involving ipsilateral nodes. All the tumors of T3 (invasion of the fascia and solitary wall effusion) and ipsilateral positive nodes (N1) are staged as IIIA, and those involving contralateral nodes (N2) are staged as IIB. Stage IV applies to the tumors with massive thoracic wall involvement (T4) and any N or M stage [10,11,12].

The fact that mesothelioma is still classified as rare cancer has contributed to slow improvements in therapeutic options in the past decades. The trimodal approach to mesothelioma treatment was introduced in the 1990s by Sugarbaker et al. [13]. The trimodal approach includes surgery (pleurectomy or extrapleural pneumonectomy—EPP), adjuvant or neoadjuvant chemotherapy, and adjuvant radiation therapy. This treatment approach resulted in median survival of 20–29 months [14,15]. The most used chemotherapy doublet in this first-line treatment is a combination of pemetrexed and cisplatin or carboplatin. Combining gemcitabine and platinum-based chemotherapy agents can also be applied with similar results [16]. The trimodal approach is still the current standard of treatment in resectable stages of mesothelioma, according to the European Society for Medical Oncology (ESMO) and NCCN guidelines [17,18]. Patients with stages I–IIIa (pleural infiltration on one side of the thorax without invasion into the thoracic wall or other structures and nodes positive only on one side) may be eligible for surgery and neoadjuvant therapy. After radical surgery, radiation therapy with up to 54–60 Gy is delivered to the whole hemithorax with newer techniques, such as Intensity Modulated Radiation Therapy (IMRT) and Image-Guided Radiation Therapy (IGRT). Adjuvant radiotherapy should, however, be delivered with great caution in patients who did not receive extended pleural pneumonectomy (EPP) since the dose to the remaining lung and other organs at risk (heart, liver, stomach) can be toxic. An EORTC multicenter phase II trial recruited 59 mesothelioma patients with stages I–III that received neoadjuvant pemetrexed/cisplatin regimen, EPP, and postoperative radiation therapy of 54 Gy in 30 fractions [19]. The trial was concluded in 2010 but failed to reach the selected time frames, and the overall survival was 18.4 months. With the ongoing debate on the safety and efficacy of postoperative radiation treatment, a reverse approach was chosen in the newly published “SMART” trial. The patients received 25 Gy in five fractions preoperatively in that trial to reduce the disease burden. The trial’s endpoint was distant recurrence which reached 63.3% at five years; however, the toxicity of this regimen has to be further evaluated [20]. Surgically operable patients can be candidates for hyperthermic intrathoracic chemotherapy (HITOCH). A systematic review by Zhao et al. suggested a more prolonged median survival of patients receiving HITOCH than patients without HITOCH. The HITOCH treatment was proposed in a palliative setting, prolonging the recurrence-free interval in these patients [21].

However, the described trimodal approach is limited to resectable stages of mesothelioma. Advanced, stage IV mesothelioma treatment can be either standard chemotherapy or the best supportive care. Still, the introduction of immunotherapy in cancer treatment has been applied to mesothelioma as well. In combination, the standard chemotherapy regimen used in the neoadjuvant setting is pemetrexed/cisplatin or carboplatin. Immunotherapy with checkpoint inhibitors nivolumab or ipilimumab can be given in first-line systemic treatment or second-line if not given prior. Pembrolizumab is also an option in second-line treatment, according to the National Comprehensive Cancer Network (NCCN) [18]. The results of immunotherapy treatment in mesothelioma are promising, with a median survival of 11.8 months for nivolumab, but we are still waiting for the overall survival data [22]. Other chemotherapeutic treatments are used in a first-line, unresectable setting. These options are a combination of pemetrexed/cisplatin/bevacizumab, gemcitabine/cisplatin, vinorelbine or, nivolumab [23]. The current standard chemotherapy treatments in mesothelioma are summarized in Table 1.

Although novel approaches for mesothelioma treatment have been extensively investigated, sixteen years have passed between the FDA approval of pemetrexed for mesothelioma treatment and the approval of a new drug for mesothelioma, namely, nivolumab/ipilimumab combination in first-line treatment [24]. Nevertheless, the usage of standard chemotherapy-based regimens (e.g., cisplatin, gemcitabine) is not obsolete in mesothelioma treatment since immunotherapy with checkpoint inhibitors (nivolumab, pembrolizumab) as standalone treatment is not the perfect solution [25,26]. Thus, a combination approach of standard treatment options and novel strategies seems feasible in mesothelioma.

**Figure 1:**
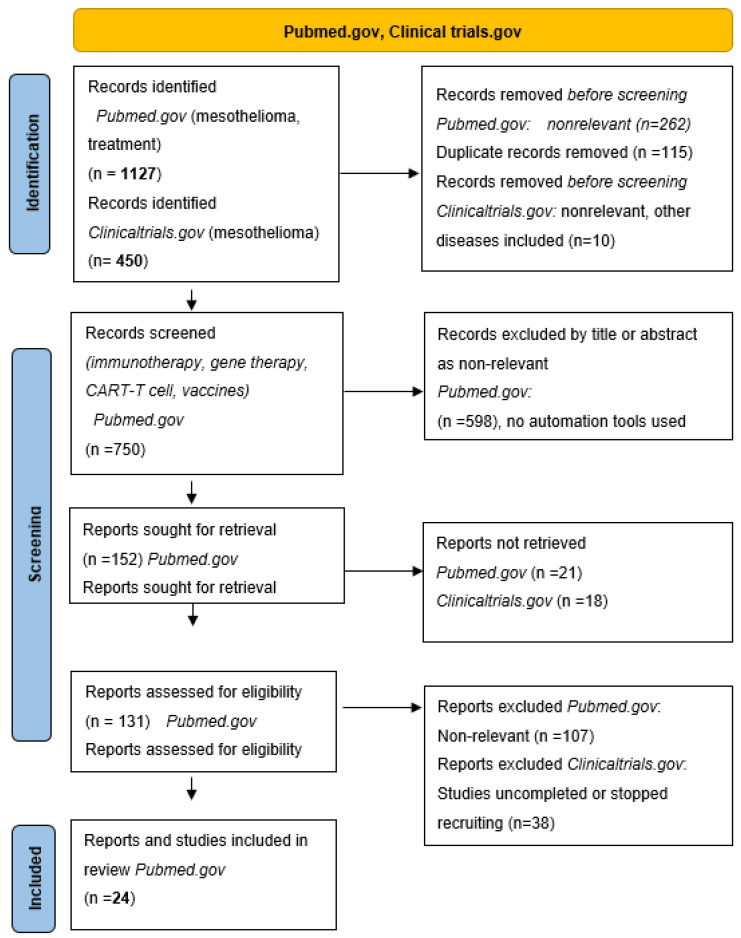
PRISMA flow diagram.

The aim of this review was to systematically summarize findings on novel treatment options in mesothelioma. We searched the available literature in the PubMed.gov and ClinicalTrials.gov databases [27,28] to systematically review the literature on novel approaches in immunotherapy, vaccines, and Chimeric Antigen Receptor (CAR)-T cell therapy in mesothelioma. We manually screened 1127 articles on PubMed and 450 trials on ClinicalTrials.gov and the retrieved papers and clinical trials exploring novel treatment options in mesothelioma were stratified into four groups: (1) Immunotherapy with checkpoint inhibitors and novel combinations with chemotherapy; (2) Oncolytic viral and vaccine therapies; (3) CAR-T cell therapy in mesothelioma, and (4) Gene and genetic therapy principles in mesothelioma. Finally, 24 papers and 12 clinical trials published in the last ten years were included in this review. A flow diagram of the systematic search for studies on novel treatments and clinical trials in mesothelioma considered in this review is shown in Figure 1.

## 2. Results and Discussion

### 2.1. Immunotherapy in Mesothelioma, Checkpoint Inhibitors and New Combinations with Chemotherapy Treatment

Immunotherapy has become another pillar of cancer treatment next to the classic chemotherapy, radiotherapy, and surgery approaches in the recent decade. Immunotherapy has been the revolutionary new therapy in lung cancer and melanoma, where treatment options were also limited. Immunotherapeutic agents are applied as first-line treatments [29,30]. However, immunotherapy is still used as a second-line treatment in mesothelioma according to the NCCN guidelines [18]. Combinations of PD1 receptor inhibitors nivolumab and pembrolizumab or monoclonal antibody ipilimumab that inhibits cytotoxic T lymphocyte protein 4 (CTLA-4) can be used in mesothelioma. The most significant benefit seemed to be from the combination of nivolumab/ipilimumab with median overall survival of 15.9 months. However, the toxicity of this combination therapy was higher (grade 3 or 4) than in the single-agent arm [31]. The CheckMate 743 trial used the combination of nivolumab/ipilimumab in the first-line setting. This study included 713 primarily unresectable mesothelioma patients and reported an overall survival advantage of 18.1 months over 14.1 months under a pemetrexed/carbo platinum regimen [32]. Another trial with pembrolizumab that included 26 patients with a performance status of WHO 0–1 and failure of previous treatment reported up to 18 months long overall survival. The overall response rate in these immunotherapy trials was around 20–30% [33]. However, when we consider potentially severe toxicities, such as pneumonitis, the results of these trials were far from miraculous [34]. Nevertheless, trials such as the CheckMate, MARS, and Keynote 028 have played an essential role in placing immunotherapy into clinical practice [31,32,33].

However, immunotherapy is not a “magical bullet” treatment in mesothelioma, and combination treatments that challenge the mechanisms of immunological resistance need to be considered. In particular, the search for novel immunotherapy agents and their application has shifted towards overcoming immunological resistance mechanisms present in mesothelioma. The answer to immunotherapy resistance in mesothelioma could be found in its complex microenvironment. Cancer-associated fibroblasts, T-cells, tumor-associated macrophages (TAMs), and myeloid-derived suppressor cells (MDSC) have immunosuppressive roles in mesothelioma. TAMs, as an example, develop an immunosuppressive phenotype in mesothelioma [35]. Furthermore, mesothelioma secretomes include granulocyte colony-stimulating factors (G-CSF) that stimulate the proliferation of MDSC, which inhibits the proliferation of T-cells [36]. 

Another more precise mechanism of immunotherapy resistance has been elucidated recently. V-domain Ig-containing suppressor of T-cell activation (VISTA) is an immune checkpoint gene that inhibits anti-tumor immune responses. The Cancer Genome Atlas Study suggested that pleural mesothelioma displays the highest expression levels of VISTA among all the cancers studied. In contrast, non-small lung cancer does not express VISTA. Therefore, VISTA has become one of the possible targets for overcoming immunotherapy resistance and a molecular target to improve the immune downregulation in mesothelioma [37,38]. 

Investigating specific and complex mesothelioma microenvironment in overcoming immunotherapy resistance gives new purpose to old chemotherapeutic agents. Cisplatin might be a promising treatment for combining immune checkpoint blocking antibodies since the studied cell lines were most susceptible to the combination treatment. It was proposed that most chemotherapy agents can enrich the microenvironment in CD3 or CD8 lymphocytes [39]. A combination of gemcitabine and anti-PD1 resulted in partial clinical response in two patients resistant to either single agent [40].

Table 2 presents an overview and more details of studies on immunotherapy in mesothelioma. However, specific immunohistological features of mesothelioma may suggest that a simple application of immune checkpoint inhibitors may not be efficient. The arising challenges in mesothelioma immunotherapy are in overcoming the immunological resistance. 

Dendritic cell therapy has been used to turn the immunologically “cold” tumor environment into an “inflamed” state in three studies that reported 20.7% overall survival at five years [41,42,43,44]. This idea of dendritic cell therapy evolved further in the DENIM trial. The patients were randomized into the dendritic cell therapy and best supportive care arm in this trial. The primary endpoint of this trial which is still recruiting, is overall survival, with secondary endpoints being progression-free survival, safety, efficacy, and quality of life. The results of this trial are still pending [45]. 

### 2.2. Therapeutic Cancer Vaccines in Mesothelioma

The modest response to immunotherapy and mesothelioma microenvironment changes warrants new approaches involving different treatment options. Oncolytic vaccines with viral vectors have been investigated in mesothelioma as a standalone treatment or in combinations involving chemo and immunotherapy. Infection with the Edmonston vaccine strain (MV-Edm) derivative of measles virus resulted in lysis of cancer cells and was tested in clinical trials for numerous tumor types, including mesothelioma. The MV-Edm receptor Cluster of Differentiation 46 (CD46) level was significantly higher in mesothelioma cells than in control cells. MV-Edm treatment of mesothelioma cell lines reduced cell viability and invoked apoptotic cell death [46]. Newer studies, however, rely on modifying the immune response. Tan et al. used an adenoviral vector to create an rAAV-soluble PD1 (sPD1)-TWIST1 vaccine that ultimately induced Twist related protein 1 (TWIST1) T-lymphocyte cell response, thereby recruiting T-cells in the murine mesothelioma model [47]. These approaches of breaking immune tolerance led to clinical trials of oncolytic vaccines. In total, 28 concluded and ongoing trials of mesothelioma vaccines are registered in ClincalTrials.gov. Table 3 summarizes the published studies on therapeutic cancer vaccines in mesothelioma.

An ongoing NIPU trial is a randomized phase II trial that included 118 patients that received prior chemotherapy and were inoperable. In one arm, patients received nivolumab and ipilimumab treatment until progression, and in the second experimental arm, the telomerase UV1 vaccine was applied in the first three months of treatment. The results are still pending [48].

The SKOPOS trial selected a combination of pemetrexed/cisplatin-based treatment with Trovax vaccine, based on a pox viral vector 5T4 tumor-associated antigen. The trial included 27 patients with locally advanced or metastatic disease who received the vaccine two weeks before chemotherapy with the combined regimen and again 24 weeks post-chemotherapy. The phase I/II study concluded with a stable disease control of 87% and adverse effects compared to chemotherapy alone [49].

Another vaccine, which is perhaps the most promising regarding mesothelioma, is a granulocyte-macrophage colony-stimulating factor (GMCSF)-expressing oncolytic adenovirus, ONCOS-102. A phase II trial with 24 included patients was concluded in May 2020 and compared oncolytic vaccine treatment ONCOS-102 and standard treatment with pemetrexed/cisplatin. The results of this trial are still pending [50].

### 2.3. CAR-T Cell Therapy in Mesothelioma

Due to the limited efficacy of checkpoint inhibitors in mesothelioma, new approaches to immunotherapy are investigated, among them chimeric antigen receptor T-cell (CAR-T) therapy. CAR-T cells are genetically engineered to recognize cancer cell-surface antigen and lyse cancer cells [51]. The target for CAR-T cells in mesothelioma and other solid cancers is mesothelin, which is abundantly expressed in mesothelioma [52,53]. Other emerging potential CAR-T targets include podoplanin (PDPN), a transmembrane receptor glycoprotein upregulated on transformed cells, cancer-associated fibroblasts, and inflammatory macrophages that contribute to cancer progression. In particular, PDPN increases tumor cell clonal capacity, epithelial–mesenchymal transition, migration, invasion, metastasis, and inflammation [54]. 

However, clinical trials are limited to mesothelin-related CAR-T cells (CAR-T cell meso). After searching the ClinicalTrials.gov website for CAR-T cell and mesothelioma, 11 trials were found. Of these trials, three were completed, one terminated, and others still ongoing. It is to be noticed that all of these trials are still in phase I [28]. The first trial included 25 mesothelioma patients with advanced disease, and CAR- T cells were injected intrapleural. The other two included patients who had pleural involvement of breast cancer. Most of the patients received pembrolizumab since the animal model studies showed that PD1 inhibition enhances CAR-T cell activity. The median overall survival in these patients was 23.9 months [55].

Another CAR-T cell meso included patients with solid cancers, including ovarian and mesothelioma. The 15 included patients were pretreated with cyclophosphamide and were chemo-refractory. CAR-T cells were applied through lentiviral transduction. This trial did not present any clinical benefit beyond stable disease, and the CAR-T cells were detectable in blood only for 28 days [56].

Table 4 summarizes the published studies on CAR-T cell therapy in mesothelioma. We may conclude from the clinical trials performed so far that CAR-T cell therapy is safe in mesothelioma. However, its efficacy needs to be further evaluated. There seem to be difficulties with the appropriate application of CAR-T cell therapy in solid tumors that need to be solved. From the trials mentioned above, we can understand that intrapleural application may be somewhat better than intravenous. The choice of viral vectors still needs to be studied further. However, the most considerable challenge in CAR-T cell therapy in mesothelioma seems to be the microenvironment that makes mesothelioma an immunologically “cold” tumor [57]. Therefore, efforts have been made in modulating this microenvironment with other agents, such as checkpoint inhibitors or classic chemotherapeutics. 

### 2.4. Gene and Genetic Therapy Principles in Mesothelioma

Therapy, based on chromosomal rearrangements, micro RNA (miRNA), short hairpin RNA (shRNA), and transcriptome technology, has been investigated on mesothelioma cell lines and animal models (Table 5). 

Chromosomal rearrangements result in novel, unique gene junctions that can be expressed and potentially result in the presentation of several neoantigens. These predicted neoantigens can be presented by tumors on major histocompatibility complex (MHC) proteins and are correlated with clonal expansion of tumor-infiltrating T cells. T cells responsive to these neoantigens have been identified in a patient’s circulation. The analysis of chromosomal rearrangements in mesothelioma can improve immunotherapeutic strategies and the selection of patients to receive immunotherapy and be applied in anti-tumor vaccines [58]. Furthermore, therapeutic principles have been studied in spliceosome’s genes—the high expression of splicing factor 3b, subunit 1 (SF3B1) correlated with poor clinical outcomes. SF3b modulators (Pladienolide-B, E7107, Meayamycin-B) showed potent cytotoxic activity in vitro [59]. 

Post-transcriptional strategies, such as RNA interference (RNAi), can have potent gene silencing in several cancers. Therefore, novel therapeutic formulations, such as DFP-1082, were developed. DFP-10825 was composed of chemically synthesized short hairpin RNA (shRNA) against thymidylate synthase (TS), a key enzyme for cancer proliferation. This therapeutic approach was studied in animal models and is now waiting for clinical trials [60,61]. 

Another post-transcriptional treatment strategy involved different micro RNAs that have been studied extensively in mesothelioma. MiRNAs can be up- or downregulated in mesothelioma tissue. An example is the miR 15/16 family that is downregulated with fibroblast growth factor (FGF). The transfection with miR 15/16 resulted in binding to the FGF receptor and diminished growth of mesothelioma cell lines [62]. 

MiR-126 was suppressed in asbestos-related malignancies. Furthermore, the application of miR-126 to the endothelial cells of mesothelioma reduced cell growth by reducing angiogenesis [63]. On the other hand, overexpression of miR-137 was linked to poor patient survival. However, the combination of miR-137 and Y-box binding protein 1 (YBX1) resulted in a tumor-suppressive function [64]. 

Other potential targets of dysregulation of miRNA in mesothelioma are miR-182/miR-183 family and miR-206. Inhibition of miR-182 and miR-183 reduced cell proliferation ability via upregulation of forkhead box 1(FOXO1) and its downstream targets, namely, p27 [65]. When miR-206 was ectopically re-expressed in mesothelioma cells and delivered to tumor xenografts in mice, it exerted significant cell killing by suppressing multiple components of the receptor-tyrosine-kinase-Ras-cell-cycle-signaling network. This miR-206-targeting mechanism manifested as induced G1/S cell cycle arrest [66].

The retrieved data indicate that the currently investigated and potential novel genetic approaches offer new possibilities of precision therapy in mesothelioma [66]. 

## 3. Methods

We searched the available literature in the PubMed.gov [27] and ClinicalTrials.gov [28] databases. The PubMed.gov search was limited to the data published from the year 2000 up to the year 2021. We used the combination of keywords “immunotherapy and mesothelioma”, “CAR-T cell therapy and mesothelioma”, “gene and genetic therapy and mesothelioma”. The ClinicalTrials.gov database search was limited to the completed studies with the keywords “mesothelioma” and “therapy”. The PRISMA flow diagram of the search is shown in Figure 1. We retrieved 24 papers from Pubmed.gov exploring the role of novel treatments in mesothelioma after manually screening 1127 records. We stratified these studies into three groups: (1) immunotherapy in mesothelioma; (2) cancer vaccines in mesothelioma; (3) CAR-T cell therapy in mesothelioma; (4) gene and genetic therapy in mesothelioma. We further searched for published and ongoing clinical trials in novel mesothelioma treatments on ClinicalTrials.gov, manually screening 450 trials and retrieving 12 trials significant to this review.

To minimize the publication bias of our systematic literature search, we have reported not only the positive sides, but also the limitations of specific therapeutic approaches. Furthermore, we also included information on clinical trials. Although in the manuscript’s text and tables we have focused on clinical trials that have published their data, extensive information on all clinical trials is available in the Supplementary Tables (Supplementary Tables S1–S3), thus minimizing any bias with regard to clinical trials. 

## 4. Conclusions

Therapeutic approaches in mesothelioma thus far have been uniform and limited to first-line, and possibly second-line, treatment. New advances in immunotherapy offer a different view on this immunologically “cold” disease. While the results of checkpoint inhibitory therapy have been modest, the research in the mesothelioma tumor microenvironment is ongoing. The combination of understanding the mesothelioma microenvironment and the genetic drivers that modulate it seems to be the future of precision-based therapy in mesothelioma.

## Figures and Tables

**Table 1:** Current standard chemotherapy treatments in mesothelioma.

Treatment Setting	Treatment Type
neoadjuvant, preoperative	pemetrexed/cisplatin, carboplatin 4–6 cycles
the first line, inoperable	pemetrexed/cisplatin, carboplatin, the addition of bevacizumab optional, 6 cycles nivolumab/ipilimumab until disease progression
second and successive treatments	gemcitabine/cisplatin, pemetrexed single, vinorelbine weekly, nivolumab/ipilimumab

Source: NCCN guidelines, Malignant pleural Mesothelioma v.2.2021 [18].

**Table 2:** Immunotherapy in mesothelioma.

Therapeutic Agent Used	Mono or Combination Therapy	Endpoints of the Study	No of the Patients Included	Significant Findings, Safety, Disease Response, Disease Control	Type of Study (Preclinical/Clinical)	Reference
Anti PD1 nivolumab and anti CTLA4 ipilimumab, as monotherapy with nivolumab or in combination	Nivolumab/ipilimumab combination	Phase II trial to assess the short-term efficacy and toxicity in a smaller group of patients	108 patients	Slightly more toxicity in the combination arm, 12-week disease control in 40% of patients in the combination arm	clinical	Scherpereel A et al. [31]
Pembrolizumab, PDL1 antibody	Monotherapy/pembro	Phase Ib trial to assess the safety and response in a smaller group of patients (25 patients)	25	Well tolerated with minimal grade III or above toxicity (4%), partial response in 25%, and stable disease in 52% of patients	clinical	Alley EW et al. [32]
Review article	Review	To review major mechanisms involved in immune resistance of mesothelioma	Review	TAM cells, dendritic cells, fibroblasts, and T-cells have a major role in immune resistance	review	Chu GJ et al. [35]
V-domain Ig-containing suppressor of T-cell activation (VISTA) as immune response inhibitor	Potential monotherapy	To study the role of VISTA in large mesothelioma samples of different mesothelioma histological types	319 mesothelioma tissue samples	VISTA is an important immune response inhibitor in mesothelioma, can be a drug target, prognostic cut off at 40%	preclinical, mesothelioma tissue lines	Muller S et al. [37]
Review of different treatment modalities	Chemotherapy, chemoradiotherapy	To review the role of standard treatments in immunomodulation of mesothelioma	8850 from 110 studies	Different, standard chemotherapy regimens can increase CD3 and CD8 lymphocytes, making mesothelioma more susceptible to immunotherapy	review	Van den Ende et al. [39]
Gemcitabine and anti PD1	Gemcitabine and anti PD1 combination	To assess gemcitabine as a potential immunomodulator	Preclinical model, 2 patients	Better tumor control and survival, nullified if dexamethasone added, clinical response in 2 patients	preclinical model and two treated patients	Tallon de L et al. [40]

**Table 3:** Overview of published studies on therapeutic cancer vaccines in mesothelioma.

Used Therapeutic Agent	Mono or Combined Therapy	Endpoints of the Study	No of the Patients Included	Major Findings, Overall Survival (OS), Disease Control, Progression-Free Survival	Type of Study (Preclinical/Clinical)	Reference
Nivolumab/Ipilimumab in one arm, telomerase UV1 vaccine in the experimental arm, second-line treatment	Combination of immunotherapy and vaccine therapy	To improve the efficacy of checkpoint inhibitors while overcoming resistance to immune therapy	118	results pending	clinical phase II study	Haakensen VD et al. [48]
Pemetrexed/Cisplatin, TroVax^®^ (pox virus viral vector)	Chemotherapy and pox virus combination	The induction of cellular or humoral anti-5T4 immune response	27	disease control in 87% of patients, overall survival 10.9 months, progression-free survival 6.8 months	clinical phase II study	Lester JF et al. [49]
GMCSF expressing oncolytic adenovirus ONCOS 102 and Pemetrexed/Cisplatin	Chemotherapy and adenovirus combination	To determine safety, response rate, overall survival, the correlation between immune activation and clinical outcome	30	completed May 2020, results pending	clinical study	Aix SP et al. [50]

**Table 4:** Overview of Chimeric Antigen Receptor (CAR)-T cell therapy in mesothelioma.

Used Therapeutic Agent	Mono or Combined Therapy	Endpoints of the Study	No of the Patients Included	Major Findings, Tolerance, Disease Progression, Disease Control	Type of Study (Preclinical/Clinical)	Reference
Anti mesothelin chimeric antigen receptor Tcell (anti-MSLN CAR-T cells)	Review	Review of phase I studies to assess the safety and efficacy of new treatment	-	Anti MSLN CAR T is safe, and efficacy is low due to mesothelioma microenvironment specifics	Clinical phase I, review	Castelletti L et al. [52]
Podoplanin, anti PDPN CAR-T cells	Preclinical	To study if this CAR-T cell can inhibit local tumor invasion and progression	preclinical	This CAR-T can be used as a biomarker or treatment target	Preclinical, cell lines	Krishnan H et al. [54]
MSLN CAR-T cells, anti PD1 pembrolizumab	Immuno/CAR-T combination	To study if this combined immunotherapy approach is safe and effective in mesothelioma patients	25	Stable disease after 6 months in 8 patients, complete radiological response in 2	Clinical phase I	Adusumilli PS et al. [55]
MSLN CAR-T cells, cyclophosphamide (a chemotherapeutic agent)	Chemotherapy/CAR-T cells	To study the safety and efficacy of this CAR-T cells chemotherapy combination	15	Well tolerated, cyclophosphamide enhances CAR-T cell expansion, low efficacy	Clinical phase I, 15 patients with mesothelioma, ovarian cancer, and pancreatic adenocarcinoma	Hass AR et al. [56]

**Table 5:** Overview of preclinical studies of genetic therapy in mesothelioma.

Used Therapeutic Agent	Endpoints of the Study	Major Findings	Type of Study	Reference
Pladienolide-B, E7107, Meayamycin-B	To study if splicing modulators can alter cell cycle and apoptosis	Splicing modulators impair mesothelioma cancer cell viability	Preclinical, mesothelioma cell lines	Sciarrillo R et al. [59]
DFP-10825 (shRNA)	To study if cationic liposomes with shRNA targeting thymidylate synthase inhibit cell growth in peritoneal mesothelioma	High therapeutic effect without severe side effects	Preclinical, mouse model	Ando H et al. [60]
miR-15/16	To study downregulation of fibroblast growth factor (FGF) by miR-15/16	miR-15/16 can downregulate FGF and inhibit the growth of mesothelioma cells	Preclinical, mesothelioma cell lines	Schelch K et al. [62]
miR-137	To study if Y box binding protein 1 gene (YBX1) can downregulate miR-137 levels in mesothelioma	miR-137 combination with YBX1 can suppress growth, invasion, and migration of mesothelioma cells	Preclinical, mesothelioma cell lines	Johnson TG et al. [64]
miR-182/miR-183	To study if inhibition of miR-182/miR-183 can reduce proliferation and migration of mesothelioma cells	miR-182/miR-183 inhibitors can reduce the proliferation and migration of mesothelioma cells	Preclinical, mesothelioma cell lines	Suzuki R et al. [65]
miR-126	To study if the re-expression of miR-126 can inhibit cell invasion and proliferation	MiR- 126 induces G1/S cell cycle arrest and inhibits proliferation	Preclinical, mesothelioma cell lines	Singh A et al. [66]

## Data Availability

All the data are presented within the article. Any additional information is available on request from the corresponding author.

## Supplementary Materials

The following supporting information can be downloaded at: https://www.mdpi.com/article/10.3390/ijms23041975/s1.
